# Supplementary material for: High-resolution whole-brain magnetic resonance spectroscopic imaging in youth at risk for psychosis
Source: Imaging Neurosci (Camb). 2026 Jun 17;4:IMAG.a.1276. doi: 10.1162/IMAG.a.1276 (PMC13277781; doi:10.1162/IMAG.a.1276)
Supplement: Supplementary Material [file IMAG.a.1276_supp.pdf]

Supplementary material

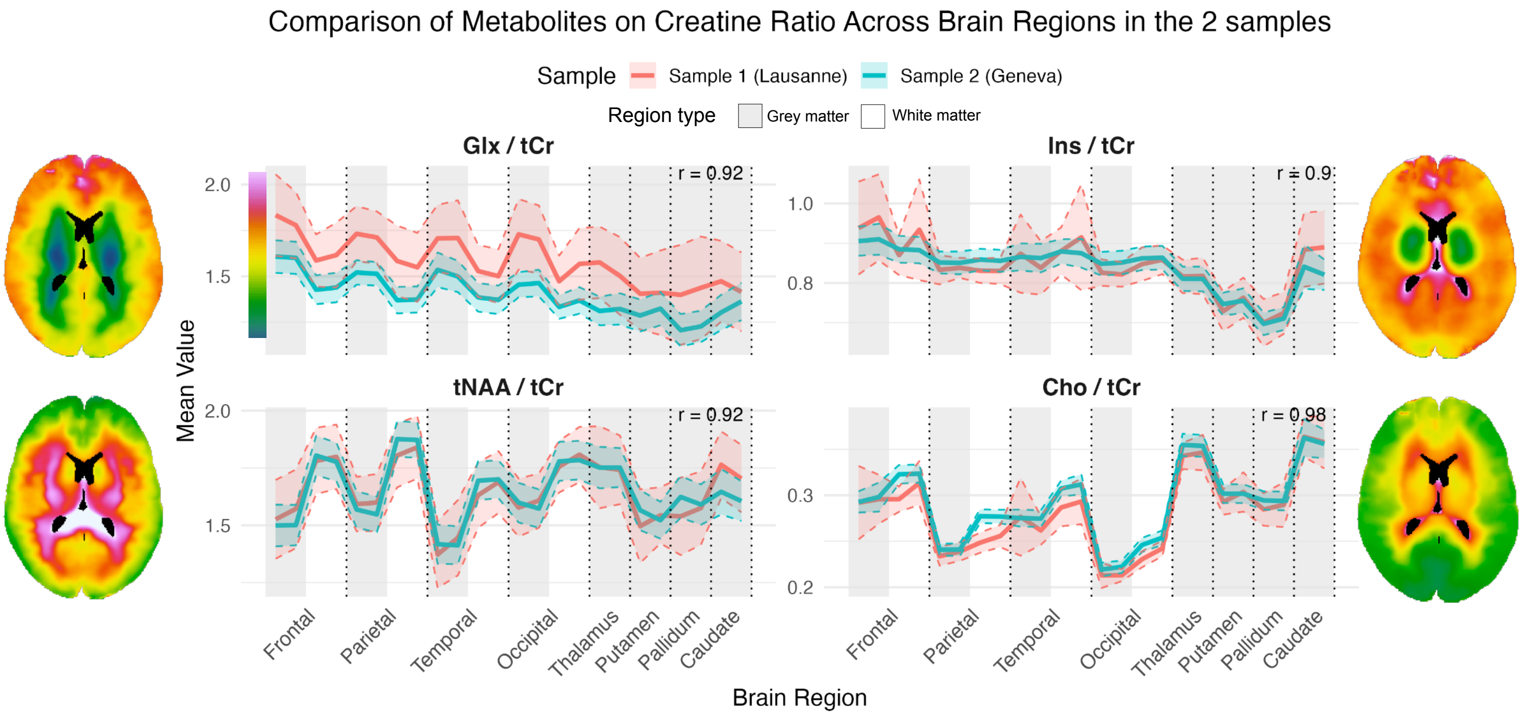

Fig. S1: Mean metabolite concentrations normalized on tCr measured across several cerebral regions (Frontal, Parietal, Temporal, Occipital, divided in grey and white matter; Thalamus, Putamen, Pallidum, Caudate) in participants from two independent samples: Lausanne (controls only, n = 13; red) and Geneva (n = 61; blue). Each region is represented by two data points corresponding to the left and right hemispheres. Correlations between the 2 samples are displayed for each metabolite or metabolite ratio plot on the top right.

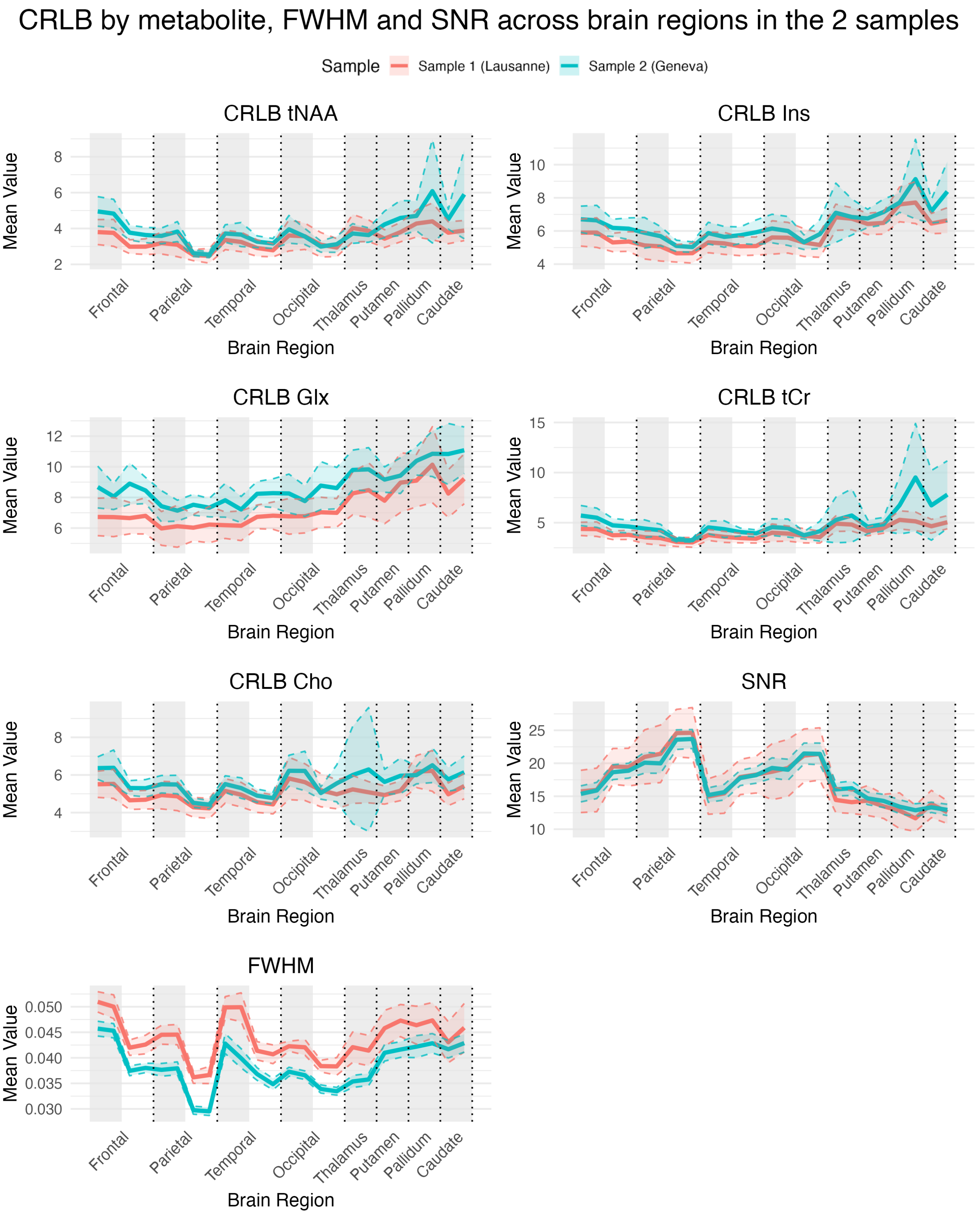

Fig. S2: Mean quality indexes (CRLB : Cramer-Rao Lower Bound ; SNR : Signal-to-Noise Ratio ; FWHM : Full Width at Half-Maximum) measured across several cerebral regions (Frontal, Parietal, Temporal, Occipital, divided in grey and white matter ; Thalamus, Putamen, Pallidum, Caudate) in participants from two independent samples : Lausanne (controls only, n = 13; red) and Geneva (n = 61; blue). Each region is represented by two data points corresponding to the left and right hemispheres.

Spectra example in one subject

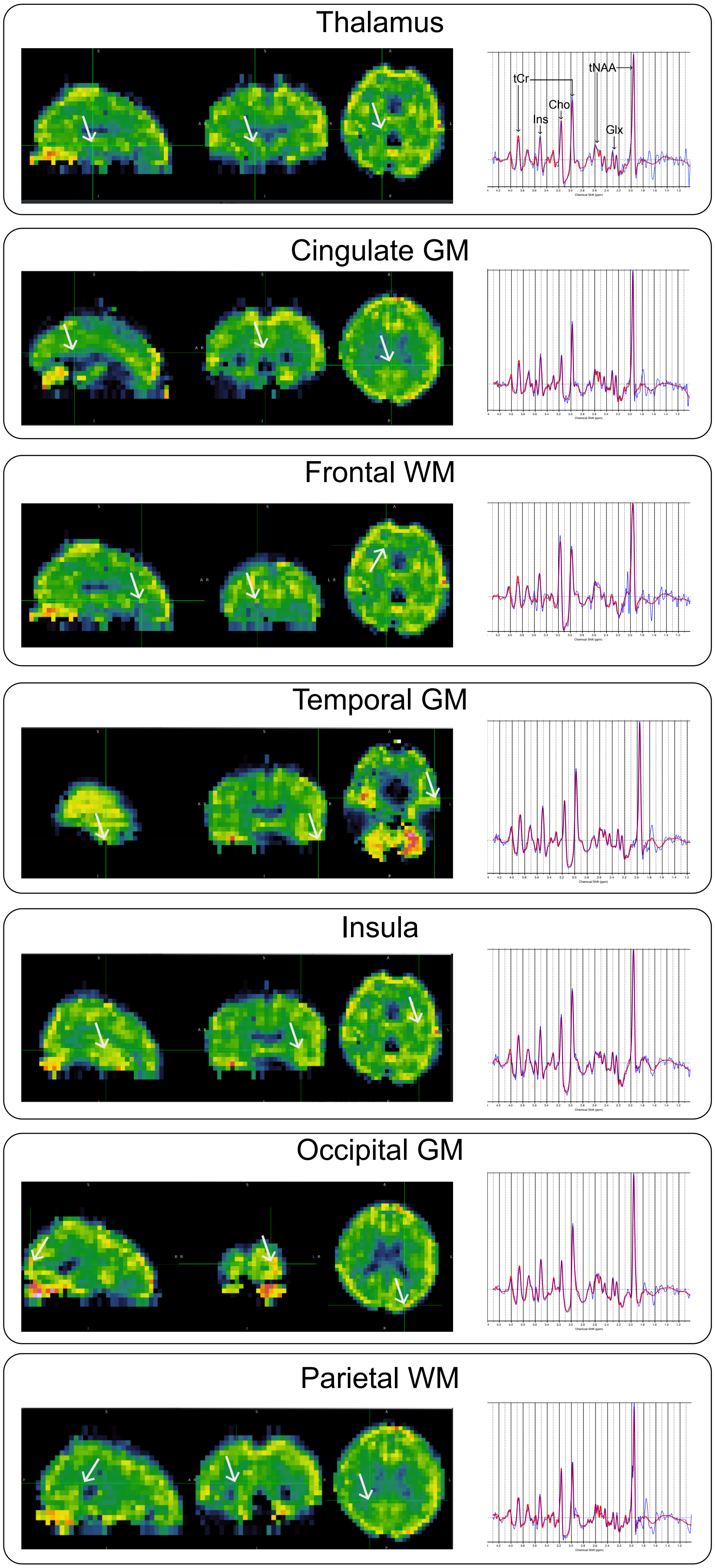

Fig. S3: An example of spectra in 7 different voxels of one control subject. Brain region is indicated based on the subject's T1w. Position of the voxel shown on the left, spectra of the given voxel on the right. The blue line corresponds to the reconstructed MRSI spectrum, the red line to the LCModel fit.

Regional coverage per metabolite

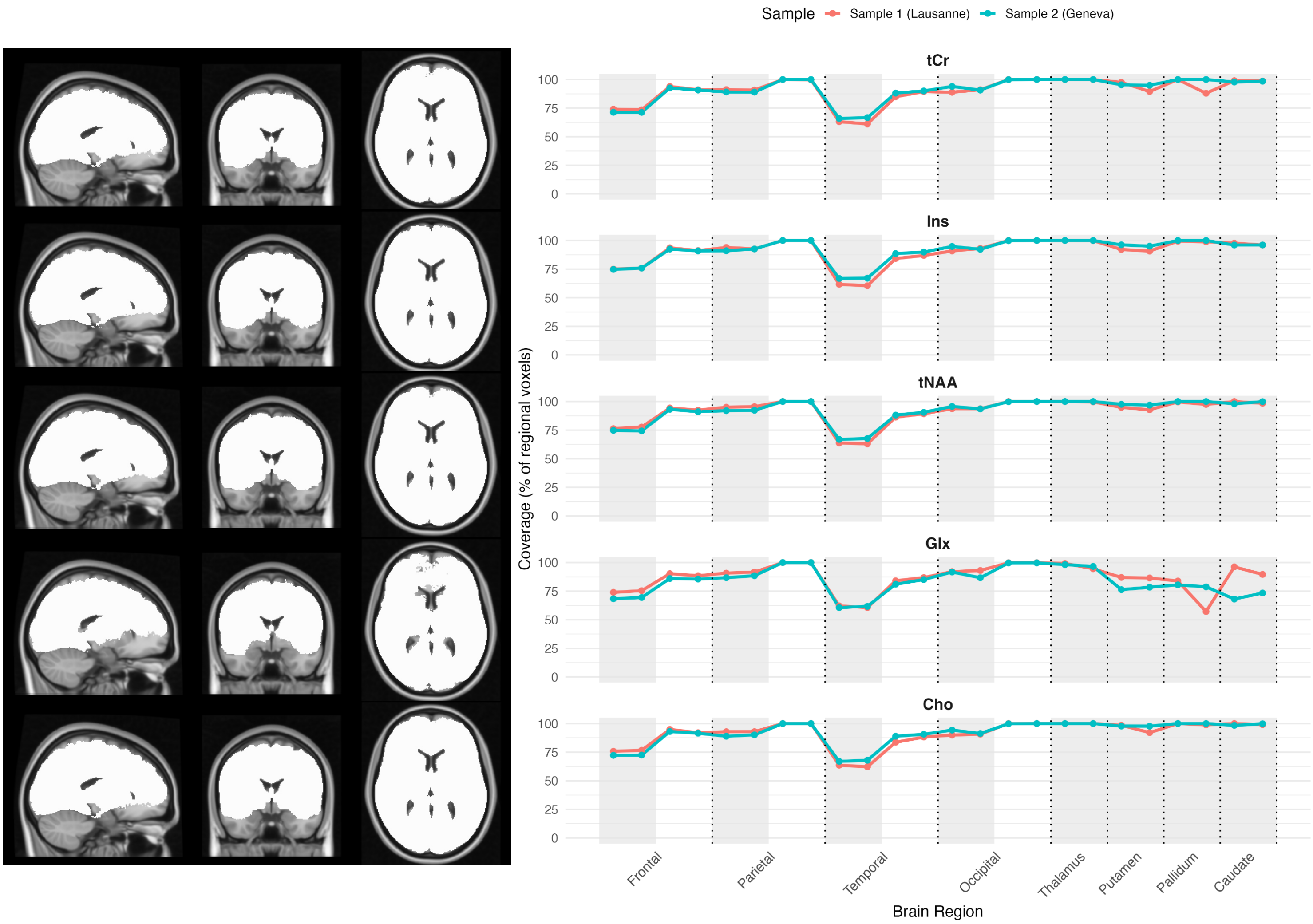

Fig. S4: Quality mask population coverage per metabolite. For each metabolite, coverage of the population is displayed in MNI (standard) space on the left. Percentage of each brain region that has good quality is displayed on the left. Frontal GM has a coverage of around 75% and temporal GM has a coverage of around 65%.

Table S1. Absolute Metabolic levels by brain regions (corrected for site)

Brain Region	Type	Side	Cohort	tCr	Cho	Glx	Ins	tNAA
Frontal	*GM*	*L*	*Sample 1 (Lausanne)*	*178.39 ± 19.32*	*44.85 ± 4.07*	*323.45 ± 72.04*	*147.02 ± 20.90*	*282.64 ± 46.95*
Frontal	*GM*	*L*	*Sample 2 (Geneva)*	*217.64 ± 32.09*	*56.08 ± 7.68*	*336.91 ± 83.91*	*184.07 ± 39.49*	*329.57 ± 71.23*
Frontal	*GM*	*R*	*Sample 1 (Lausanne)*	*185.41 ± 19.85*	*46.68 ± 4.68*	*336.91 ± 77.59*	*151.48 ± 21.94*	*285.03 ± 46.55*
Frontal	*GM*	*R*	*Sample 2 (Geneva)*	*214.50 ± 35.12*	*55.82 ± 8.69*	*332.75 ± 89.74*	*182.78 ± 40.77*	*327.56 ± 76.42*
Frontal	*WM*	*L*	*Sample 1 (Lausanne)*	*200.15 ± 20.00*	*57.76 ± 5.57*	*330.81 ± 82.65*	*169.31 ± 20.96*	*371.07 ± 43.07*
Frontal	*WM*	*L*	*Sample 2 (Geneva)*	*219.24 ± 27.16*	*66.93 ± 7.69*	*312.17 ± 80.52*	*189.81 ± 36.73*	*399.14 ± 77.01*
Frontal	*WM*	*R*	*Sample 1 (Lausanne)*	*203.51 ± 22.67*	*57.76 ± 6.73*	*339.88 ± 88.38*	*171.18 ± 23.25*	*370.22 ± 41.74*
Frontal	*WM*	*R*	*Sample 2 (Geneva)*	*216.39 ± 27.41*	*66.60 ± 8.34*	*311.43 ± 84.02*	*187.97 ± 36.88*	*397.51 ± 77.31*
Parietal	*GM*	*L*	*Sample 1 (Lausanne)*	*192.43 ± 22.99*	*43.85 ± 5.26*	*328.92 ± 74.74*	*153.77 ± 23.48*	*303.82 ± 49.33*
Parietal	*GM*	*L*	*Sample 2 (Geneva)*	*220.16 ± 33.58*	*51.35 ± 7.25*	*334.35 ± 84.76*	*185.09 ± 39.35*	*335.56 ± 69.36*
Parietal	*GM*	*R*	*Sample 1 (Lausanne)*	*186.38 ± 18.14*	*42.71 ± 4.33*	*323.72 ± 67.07*	*147.79 ± 18.75*	*285.47 ± 40.40*
Parietal	*GM*	*R*	*Sample 2 (Geneva)*	*216.03 ± 33.93*	*50.85 ± 7.27*	*330.00 ± 89.13*	*183.20 ± 38.94*	*334.33 ± 71.77*
Parietal	*WM*	*L*	*Sample 1 (Lausanne)*	*210.23 ± 23.46*	*53.15 ± 5.80*	*324.11 ± 77.58*	*174.32 ± 24.49*	*382.52 ± 43.20*

Brain Region	Type	Side	Cohort	tCr	Cho	Glx	Ins	tNAA
Parietal	*WM*	*L*	*Sample 2 (Geneva)*	*225.08 ± 26.67*	*61.21 ± 6.86*	*312.92 ± 81.94*	*193.26 ± 35.59*	*415.48 ± 71.12*
Parietal	*WM*	*R*	*Sample 1 (Lausanne)*	*203.11 ± 24.36*	*49.93 ± 6.07*	*320.97 ± 76.01*	*168.51 ± 23.87*	*360.31 ± 39.14*
Parietal	*WM*	*R*	*Sample 2 (Geneva)*	*216.62 ± 25.60*	*59.07 ± 6.84*	*300.91 ± 81.80*	*186.74 ± 34.16*	*403.38 ± 70.50*
Temporal	*GM*	*L*	*Sample 1 (Lausanne)*	*210.41 ± 17.05*	*50.93 ± 4.62*	*364.71 ± 67.12*	*171.81 ± 19.34*	*321.97 ± 36.04*
Temporal	*GM*	*L*	*Sample 2 (Geneva)*	*225.98 ± 30.98*	*57.16 ± 6.87*	*353.88 ± 92.96*	*195.19 ± 38.77*	*336.21 ± 65.39*
Temporal	*GM*	*R*	*Sample 1 (Lausanne)*	*205.04 ± 15.98*	*47.67 ± 4.15*	*357.06 ± 61.88*	*166.51 ± 17.81*	*305.05 ± 43.56*
Temporal	*GM*	*R*	*Sample 2 (Geneva)*	*222.09 ± 29.93*	*56.12 ± 6.65*	*347.56 ± 95.05*	*190.68 ± 37.55*	*332.46 ± 68.56*
Temporal	*WM*	*L*	*Sample 1 (Lausanne)*	*197.42 ± 18.74*	*54.70 ± 5.59*	*304.91 ± 70.34*	*166.75 ± 19.91*	*354.33 ± 26.94*
Temporal	*WM*	*L*	*Sample 2 (Geneva)*	*212.55 ± 26.32*	*63.52 ± 6.67*	*301.03 ± 82.00*	*185.45 ± 35.56*	*374.65 ± 66.84*
Temporal	*WM*	*R*	*Sample 1 (Lausanne)*	*193.42 ± 15.68*	*51.81 ± 5.44*	*303.41 ± 68.78*	*163.32 ± 19.53*	*334.44 ± 35.39*
Temporal	*WM*	*R*	*Sample 2 (Geneva)*	*206.13 ± 23.87*	*61.01 ± 6.22*	*290.95 ± 80.41*	*180.37 ± 33.51*	*366.24 ± 67.91*
Occipital	*GM*	*L*	*Sample 1 (Lausanne)*	*205.58 ± 16.06*	*43.18 ± 3.78*	*334.88 ± 64.29*	*166.89 ± 16.08*	*323.70 ± 35.12*
Occipital	*GM*	*L*	*Sample 2 (Geneva)*	*223.97 ± 32.29*	*49.43 ± 5.89*	*333.31 ± 87.29*	*190.68 ± 38.71*	*350.08 ± 73.27*
Occipital	*GM*	*R*	*Sample 1 (Lausanne)*	*196.15 ± 15.72*	*39.92 ± 3.87*	*322.81 ± 60.49*	*161.15 ± 17.72*	*304.47 ± 42.54*

Brain Region	Type	Side	Cohort	tCr	Cho	Glx	Ins	tNAA
Occipital	*GM*	*R*	*Sample 2 (Geneva)*	*218.29 ± 30.45*	*46.63 ± 5.14*	*324.20 ± 87.30*	*184.56 ± 37.09*	*341.57 ± 70.54*
Occipital	*WM*	*L*	*Sample 1 (Lausanne)*	*204.50 ± 16.73*	*48.16 ± 5.12*	*317.93 ± 65.13*	*174.13 ± 19.78*	*362.84 ± 33.62*
Occipital	*WM*	*L*	*Sample 2 (Geneva)*	*222.17 ± 28.44*	*55.13 ± 5.86*	*310.49 ± 83.36*	*192.29 ± 37.54*	*393.69 ± 77.14*
Occipital	*WM*	*R*	*Sample 1 (Lausanne)*	*196.95 ± 14.48*	*44.44 ± 4.84*	*306.07 ± 70.79*	*165.48 ± 20.19*	*341.27 ± 43.49*
Occipital	*WM*	*R*	*Sample 2 (Geneva)*	*217.06 ± 27.04*	*52.16 ± 5.09*	*300.72 ± 84.01*	*187.58 ± 36.37*	*385.83 ± 73.18*
Thalamus	*GM*	*L*	*Sample 1 (Lausanne)*	*155.28 ± 25.19*	*50.40 ± 8.23*	*266.94 ± 98.78*	*122.08 ± 20.27*	*255.67 ± 36.75*
Thalamus	*GM*	*L*	*Sample 2 (Geneva)*	*183.59 ± 28.99*	*62.55 ± 9.04*	*254.40 ± 75.29*	*146.66 ± 30.49*	*312.36 ± 64.70*
Thalamus	*GM*	*R*	*Sample 1 (Lausanne)*	*152.98 ± 23.08*	*50.74 ± 8.55*	*257.21 ± 94.62*	*123.04 ± 19.80*	*257.98 ± 48.22*
Thalamus	*GM*	*R*	*Sample 2 (Geneva)*	*181.19 ± 27.59*	*62.02 ± 8.92*	*259.45 ± 75.49*	*145.00 ± 29.39*	*307.10 ± 62.87*
Putamen	*GM*	*L*	*Sample 1 (Lausanne)*	*190.52 ± 27.98*	*57.67 ± 9.63*	*298.10 ± 113.82*	*142.50 ± 24.69*	*307.61 ± 39.69*
Putamen	*GM*	*L*	*Sample 2 (Geneva)*	*221.59 ± 36.01*	*64.36 ± 10.17*	*283.20 ± 94.01*	*163.60 ± 35.96*	*343.00 ± 75.13*
Putamen	*GM*	*R*	*Sample 1 (Lausanne)*	*200.21 ± 30.08*	*58.63 ± 9.16*	*299.70 ± 102.73*	*142.01 ± 21.42*	*316.07 ± 46.84*
Putamen	*GM*	*R*	*Sample 2 (Geneva)*	*222.41 ± 31.51*	*65.23 ± 9.02*	*280.46 ± 91.67*	*162.54 ± 31.05*	*347.28 ± 71.69*
Pallidum	*GM*	*L*	*Sample 1 (Lausanne)*	*175.92 ± 31.99*	*50.66 ± 11.48*	*277.16 ± 106.45*	*128.79 ± 28.20*	*287.19 ± 45.43*

Brain Region	Type	Side	Cohort	tCr	Cho	Glx	Ins	tNAA
Pallidum	*GM*	*L*	*Sample 2 (Geneva)*	*196.95 ± 37.52*	*56.33 ± 10.51*	*248.62 ± 85.23*	*137.69 ± 32.37*	*313.46 ± 81.46*
Pallidum	*GM*	*R*	*Sample 1 (Lausanne)*	*177.46 ± 39.89*	*50.05 ± 11.20*	*250.81 ± 95.30*	*125.39 ± 27.27*	*279.54 ± 54.65*
Pallidum	*GM*	*R*	*Sample 2 (Geneva)*	*197.62 ± 34.96*	*56.92 ± 10.27*	*243.32 ± 92.00*	*136.83 ± 30.95*	*317.13 ± 80.47*
Caudate	*GM*	*L*	*Sample 1 (Lausanne)*	*147.73 ± 25.39*	*52.38 ± 8.00*	*210.12 ± 79.21*	*130.68 ± 24.08*	*264.74 ± 40.58*
Caudate	*GM*	*L*	*Sample 2 (Geneva)*	*177.73 ± 30.25*	*60.65 ± 10.46*	*242.94 ± 84.53*	*145.28 ± 32.70*	*285.40 ± 65.49*
Caudate	*GM*	*R*	*Sample 1 (Lausanne)*	*154.98 ± 25.16*	*55.70 ± 7.50*	*230.46 ± 73.17*	*134.53 ± 20.64*	*276.67 ± 46.50*
Caudate	*GM*	*R*	*Sample 2 (Geneva)*	*180.80 ± 30.02*	*63.09 ± 9.84*	*230.17 ± 66.48*	*148.25 ± 30.41*	*299.67 ± 63.27*

Table S2. Ratio of metabolite / sum of 5 metabolites by brain region

Brain Region	Type	Side	Cohort	tCr / sum	Cho / sum	Glx / sum	Ins / sum	tNAA / sum
Frontal	*GM*	*L*	*Sample 1 (Lausanne)*	*0.18 ± 0.02*	*0.05 ± 0.00*	*0.33 ± 0.04*	*0.15 ± 0.01*	*0.29 ± 0.03*
Frontal	*GM*	*L*	*Sample 2 (Geneva)*	*0.19 ± 0.02*	*0.05 ± 0.00*	*0.29 ± 0.04*	*0.17 ± 0.02*	*0.30 ± 0.04*
Frontal	*GM*	*R*	*Sample 1 (Lausanne)*	*0.18 ± 0.02*	*0.05 ± 0.00*	*0.33 ± 0.04*	*0.15 ± 0.01*	*0.29 ± 0.03*
Frontal	*GM*	*R*	*Sample 2 (Geneva)*	*0.19 ± 0.02*	*0.05 ± 0.00*	*0.29 ± 0.04*	*0.17 ± 0.02*	*0.30 ± 0.04*
Frontal	*WM*	*L*	*Sample 1 (Lausanne)*	*0.18 ± 0.01*	*0.05 ± 0.01*	*0.29 ± 0.04*	*0.15 ± 0.01*	*0.33 ± 0.03*
Frontal	*WM*	*L*	*Sample 2 (Geneva)*	*0.19 ± 0.02*	*0.06 ± 0.01*	*0.26 ± 0.03*	*0.16 ± 0.02*	*0.34 ± 0.04*
Frontal	*WM*	*R*	*Sample 1 (Lausanne)*	*0.18 ± 0.01*	*0.05 ± 0.01*	*0.29 ± 0.04*	*0.15 ± 0.01*	*0.33 ± 0.03*
Frontal	*WM*	*R*	*Sample 2 (Geneva)*	*0.19 ± 0.02*	*0.06 ± 0.01*	*0.26 ± 0.04*	*0.16 ± 0.02*	*0.34 ± 0.04*
Parietal	*GM*	*L*	*Sample 1 (Lausanne)*	*0.19 ± 0.02*	*0.04 ± 0.00*	*0.32 ± 0.04*	*0.15 ± 0.01*	*0.30 ± 0.03*
Parietal	*GM*	*L*	*Sample 2 (Geneva)*	*0.20 ± 0.02*	*0.05 ± 0.00*	*0.29 ± 0.04*	*0.17 ± 0.02*	*0.30 ± 0.04*
Parietal	*GM*	*R*	*Sample 1 (Lausanne)*	*0.19 ± 0.02*	*0.04 ± 0.00*	*0.32 ± 0.04*	*0.15 ± 0.01*	*0.29 ± 0.03*
Parietal	*GM*	*R*	*Sample 2 (Geneva)*	*0.19 ± 0.02*	*0.05 ± 0.00*	*0.29 ± 0.04*	*0.17 ± 0.02*	*0.30 ± 0.04*
Parietal	*WM*	*L*	*Sample 1 (Lausanne)*	*0.18 ± 0.02*	*0.05 ± 0.01*	*0.28 ± 0.04*	*0.15 ± 0.01*	*0.34 ± 0.03*

**Brain Region**	**Type**	**Side**	**Cohort**	**tCr / sum**	**Cho / sum**	**Glx / sum**	**Ins / sum**	**tNAA / sum**
Parietal	*WM*	*L*	*Sample 2 (Geneva)*	*0.19 ± 0.02*	*0.05 ± 0.01*	*0.25 ± 0.04*	*0.16 ± 0.02*	*0.35 ± 0.04*
Parietal	*WM*	*R*	*Sample 1 (Lausanne)*	*0.18 ± 0.02*	*0.05 ± 0.00*	*0.29 ± 0.04*	*0.15 ± 0.01*	*0.33 ± 0.03*
Parietal	*WM*	*R*	*Sample 2 (Geneva)*	*0.19 ± 0.02*	*0.05 ± 0.01*	*0.25 ± 0.04*	*0.16 ± 0.02*	*0.35 ± 0.04*
Temporal	*GM*	*L*	*Sample 1 (Lausanne)*	*0.19 ± 0.02*	*0.05 ± 0.00*	*0.32 ± 0.04*	*0.15 ± 0.01*	*0.29 ± 0.03*
Temporal	*GM*	*L*	*Sample 2 (Geneva)*	*0.20 ± 0.02*	*0.05 ± 0.01*	*0.29 ± 0.04*	*0.17 ± 0.02*	*0.29 ± 0.04*
Temporal	*GM*	*R*	*Sample 1 (Lausanne)*	*0.19 ± 0.02*	*0.04 ± 0.00*	*0.33 ± 0.04*	*0.15 ± 0.01*	*0.28 ± 0.03*
Temporal	*GM*	*R*	*Sample 2 (Geneva)*	*0.20 ± 0.02*	*0.05 ± 0.01*	*0.29 ± 0.04*	*0.17 ± 0.02*	*0.29 ± 0.04*
Temporal	*WM*	*L*	*Sample 1 (Lausanne)*	*0.18 ± 0.01*	*0.05 ± 0.01*	*0.28 ± 0.05*	*0.16 ± 0.01*	*0.33 ± 0.03*
Temporal	*WM*	*L*	*Sample 2 (Geneva)*	*0.19 ± 0.02*	*0.06 ± 0.01*	*0.25 ± 0.04*	*0.16 ± 0.02*	*0.33 ± 0.04*
Temporal	*WM*	*R*	*Sample 1 (Lausanne)*	*0.19 ± 0.01*	*0.05 ± 0.01*	*0.28 ± 0.05*	*0.16 ± 0.01*	*0.32 ± 0.04*
Temporal	*WM*	*R*	*Sample 2 (Geneva)*	*0.19 ± 0.02*	*0.06 ± 0.01*	*0.25 ± 0.04*	*0.17 ± 0.02*	*0.33 ± 0.04*
Occipital	*GM*	*L*	*Sample 1 (Lausanne)*	*0.19 ± 0.01*	*0.04 ± 0.00*	*0.31 ± 0.04*	*0.15 ± 0.01*	*0.31 ± 0.03*
Occipital	*GM*	*L*	*Sample 2 (Geneva)*	*0.20 ± 0.02*	*0.04 ± 0.00*	*0.28 ± 0.04*	*0.17 ± 0.02*	*0.31 ± 0.04*
Occipital	*GM*	*R*	*Sample 1 (Lausanne)*	*0.19 ± 0.01*	*0.04 ± 0.00*	*0.31 ± 0.04*	*0.16 ± 0.01*	*0.30 ± 0.03*

Brain Region	Type	Side	Cohort	tCr / sum	Cho / sum	Glx / sum	Ins / sum	tNAA / sum
Occipital	*GM*	*R*	*Sample 2 (Geneva)*	*0.20 ± 0.02*	*0.04 ± 0.00*	*0.28 ± 0.04*	*0.17 ± 0.02*	*0.31 ± 0.04*
Occipital	*WM*	*L*	*Sample 1 (Lausanne)*	*0.18 ± 0.01*	*0.04 ± 0.00*	*0.28 ± 0.04*	*0.16 ± 0.01*	*0.33 ± 0.03*
Occipital	*WM*	*L*	*Sample 2 (Geneva)*	*0.19 ± 0.02*	*0.05 ± 0.01*	*0.26 ± 0.04*	*0.16 ± 0.02*	*0.34 ± 0.04*
Occipital	*WM*	*R*	*Sample 1 (Lausanne)*	*0.19 ± 0.01*	*0.04 ± 0.00*	*0.29 ± 0.05*	*0.16 ± 0.01*	*0.33 ± 0.04*
Occipital	*WM*	*R*	*Sample 2 (Geneva)*	*0.19 ± 0.02*	*0.05 ± 0.01*	*0.26 ± 0.04*	*0.17 ± 0.02*	*0.34 ± 0.05*
Thalamus	*GM*	*L*	*Sample 1 (Lausanne)*	*0.19 ± 0.02*	*0.06 ± 0.01*	*0.28 ± 0.06*	*0.15 ± 0.01*	*0.32 ± 0.05*
Thalamus	*GM*	*L*	*Sample 2 (Geneva)*	*0.19 ± 0.02*	*0.07 ± 0.01*	*0.25 ± 0.04*	*0.16 ± 0.02*	*0.33 ± 0.04*
Thalamus	*GM*	*R*	*Sample 1 (Lausanne)*	*0.18 ± 0.01*	*0.06 ± 0.01*	*0.30 ± 0.07*	*0.15 ± 0.01*	*0.31 ± 0.05*
Thalamus	*GM*	*R*	*Sample 2 (Geneva)*	*0.19 ± 0.02*	*0.07 ± 0.01*	*0.25 ± 0.04*	*0.16 ± 0.02*	*0.33 ± 0.05*
Putamen	*GM*	*L*	*Sample 1 (Lausanne)*	*0.20 ± 0.02*	*0.06 ± 0.01*	*0.29 ± 0.07*	*0.14 ± 0.01*	*0.32 ± 0.04*
Putamen	*GM*	*L*	*Sample 2 (Geneva)*	*0.21 ± 0.02*	*0.06 ± 0.01*	*0.25 ± 0.04*	*0.15 ± 0.02*	*0.32 ± 0.04*
Putamen	*GM*	*R*	*Sample 1 (Lausanne)*	*0.20 ± 0.02*	*0.06 ± 0.01*	*0.29 ± 0.06*	*0.14 ± 0.01*	*0.31 ± 0.05*
Putamen	*GM*	*R*	*Sample 2 (Geneva)*	*0.21 ± 0.03*	*0.06 ± 0.01*	*0.25 ± 0.05*	*0.15 ± 0.02*	*0.32 ± 0.05*
Pallidum	*GM*	*L*	*Sample 1 (Lausanne)*	*0.20 ± 0.02*	*0.06 ± 0.01*	*0.30 ± 0.08*	*0.14 ± 0.01*	*0.33 ± 0.05*

**Brain Region**	**Type**	**Side**	**Cohort**	**tCr / sum**	**Cho / sum**	**Glx / sum**	**Ins / sum**	**tNAA / sum**
Pallidum	*GM*	*L*	*Sample 2 (Geneva)*	*0.21 ± 0.03*	*0.06 ± 0.01*	*0.24 ± 0.04*	*0.15 ± 0.02*	*0.33 ± 0.05*
Pallidum	*GM*	*R*	*Sample 1 (Lausanne)*	*0.21 ± 0.02*	*0.06 ± 0.01*	*0.27 ± 0.06*	*0.14 ± 0.01*	*0.32 ± 0.05*
Pallidum	*GM*	*R*	*Sample 2 (Geneva)*	*0.21 ± 0.03*	*0.06 ± 0.01*	*0.24 ± 0.05*	*0.14 ± 0.02*	*0.33 ± 0.05*
Caudate	*GM*	*L*	*Sample 1 (Lausanne)*	*0.19 ± 0.02*	*0.07 ± 0.01*	*0.25 ± 0.05*	*0.16 ± 0.02*	*0.33 ± 0.04*
Caudate	*GM*	*L*	*Sample 2 (Geneva)*	*0.20 ± 0.03*	*0.07 ± 0.01*	*0.25 ± 0.05*	*0.16 ± 0.03*	*0.32 ± 0.05*
Caudate	*GM*	*R*	*Sample 1 (Lausanne)*	*0.18 ± 0.02*	*0.07 ± 0.01*	*0.26 ± 0.05*	*0.16 ± 0.02*	*0.32 ± 0.04*
Caudate	*GM*	*R*	*Sample 2 (Geneva)*	*0.20 ± 0.03*	*0.07 ± 0.01*	*0.24 ± 0.04*	*0.16 ± 0.02*	*0.32 ± 0.05*

Table S3. Ratio of metabolite / creatine by brain region

Brain region	Type	Side	Cohort	Cho / tCr	Glx / tCr	Ins / tCr	tNAA / tCr
Frontal	*GM*	*L*	*Sample 1 (Lausanne)*	*0.30 ± 0.04*	*1.78 ± 0.25*	*0.96 ± 0.17*	*1.57 ± 0.27*
Frontal	*GM*	*L*	*Sample 2 (Geneva)*	*0.30 ± 0.06*	*1.60 ± 0.33*	*0.91 ± 0.15*	*1.50 ± 0.34*
Frontal	*GM*	*R*	*Sample 1 (Lausanne)*	*0.29 ± 0.06*	*1.83 ± 0.33*	*0.94 ± 0.18*	*1.53 ± 0.27*
Frontal	*GM*	*R*	*Sample 2 (Geneva)*	*0.29 ± 0.04*	*1.61 ± 0.34*	*0.91 ± 0.14*	*1.50 ± 0.35*
Frontal	*WM*	*L*	*Sample 1 (Lausanne)*	*0.31 ± 0.04*	*1.62 ± 0.28*	*0.93 ± 0.21*	*1.80 ± 0.23*
Frontal	*WM*	*L*	*Sample 2 (Geneva)*	*0.32 ± 0.04*	*1.44 ± 0.31*	*0.88 ± 0.13*	*1.78 ± 0.33*
Frontal	*WM*	*R*	*Sample 1 (Lausanne)*	*0.30 ± 0.02*	*1.59 ± 0.22*	*0.87 ± 0.08*	*1.78 ± 0.24*
Frontal	*WM*	*R*	*Sample 2 (Geneva)*	*0.32 ± 0.04*	*1.43 ± 0.30*	*0.88 ± 0.13*	*1.80 ± 0.34*
Parietal	*GM*	*L*	*Sample 1 (Lausanne)*	*0.24 ± 0.02*	*1.71 ± 0.22*	*0.84 ± 0.04*	*1.60 ± 0.21*
Parietal	*GM*	*L*	*Sample 2 (Geneva)*	*0.24 ± 0.03*	*1.51 ± 0.25*	*0.85 ± 0.11*	*1.55 ± 0.30*
Parietal	*GM*	*R*	*Sample 1 (Lausanne)*	*0.23 ± 0.02*	*1.73 ± 0.24*	*0.83 ± 0.06*	*1.59 ± 0.20*
Parietal	*GM*	*R*	*Sample 2 (Geneva)*	*0.24 ± 0.02*	*1.52 ± 0.26*	*0.85 ± 0.11*	*1.57 ± 0.31*
Parietal	*WM*	*L*	*Sample 1 (Lausanne)*	*0.26 ± 0.02*	*1.55 ± 0.31*	*0.83 ± 0.05*	*1.84 ± 0.23*
Parietal	*WM*	*L*	*Sample 2 (Geneva)*	*0.28 ± 0.03*	*1.37 ± 0.28*	*0.86 ± 0.11*	*1.87 ± 0.30*

Brain region	Type	Side	Cohort	Cho / tCr	Glx / tCr	Ins / tCr	tNAA / tCr
Parietal	*WM*	*R*	*Sample 1 (Lausanne)*	*0.25 ± 0.02*	*1.58 ± 0.32*	*0.83 ± 0.05*	*1.80 ± 0.23*
Parietal	*WM*	*R*	*Sample 2 (Geneva)*	*0.28 ± 0.03*	*1.37 ± 0.28*	*0.86 ± 0.11*	*1.88 ± 0.30*
Temporal	*GM*	*L*	*Sample 1 (Lausanne)*	*0.26 ± 0.02*	*1.71 ± 0.29*	*0.84 ± 0.09*	*1.45 ± 0.23*
Temporal	*GM*	*L*	*Sample 2 (Geneva)*	*0.27 ± 0.04*	*1.50 ± 0.33*	*0.86 ± 0.14*	*1.41 ± 0.32*
Temporal	*GM*	*R*	*Sample 1 (Lausanne)*	*0.28 ± 0.06*	*1.71 ± 0.24*	*0.87 ± 0.14*	*1.37 ± 0.20*
Temporal	*GM*	*R*	*Sample 2 (Geneva)*	*0.28 ± 0.04*	*1.54 ± 0.36*	*0.87 ± 0.13*	*1.42 ± 0.33*
Temporal	*WM*	*L*	*Sample 1 (Lausanne)*	*0.29 ± 0.04*	*1.50 ± 0.22*	*0.91 ± 0.21*	*1.69 ± 0.21*
Temporal	*WM*	*L*	*Sample 2 (Geneva)*	*0.31 ± 0.04*	*1.37 ± 0.30*	*0.87 ± 0.14*	*1.70 ± 0.32*
Temporal	*WM*	*R*	*Sample 1 (Lausanne)*	*0.29 ± 0.03*	*1.53 ± 0.21*	*0.88 ± 0.09*	*1.63 ± 0.22*
Temporal	*WM*	*R*	*Sample 2 (Geneva)*	*0.31 ± 0.03*	*1.38 ± 0.30*	*0.88 ± 0.12*	*1.69 ± 0.33*
Occipital	*GM*	*L*	*Sample 1 (Lausanne)*	*0.21 ± 0.01*	*1.70 ± 0.31*	*0.82 ± 0.05*	*1.61 ± 0.20*
Occipital	*GM*	*L*	*Sample 2 (Geneva)*	*0.22 ± 0.03*	*1.46 ± 0.28*	*0.85 ± 0.12*	*1.57 ± 0.33*
Occipital	*GM*	*R*	*Sample 1 (Lausanne)*	*0.21 ± 0.02*	*1.73 ± 0.32*	*0.83 ± 0.05*	*1.58 ± 0.21*
Occipital	*GM*	*R*	*Sample 2 (Geneva)*	*0.22 ± 0.03*	*1.45 ± 0.26*	*0.85 ± 0.12*	*1.60 ± 0.34*

Brain region	Type	Side	Cohort	Cho / tCr	Glx / tCr	Ins / tCr	tNAA / tCr
Occipital	*WM*	*L*	*Sample 1 (Lausanne)*	*0.24 ± 0.01*	*1.57 ± 0.32*	*0.86 ± 0.06*	*1.81 ± 0.20*
Occipital	*WM*	*L*	*Sample 2 (Geneva)*	*0.25 ± 0.03*	*1.37 ± 0.28*	*0.86 ± 0.12*	*1.78 ± 0.33*
Occipital	*WM*	*R*	*Sample 1 (Lausanne)*	*0.23 ± 0.01*	*1.47 ± 0.22*	*0.85 ± 0.07*	*1.76 ± 0.19*
Occipital	*WM*	*R*	*Sample 2 (Geneva)*	*0.25 ± 0.03*	*1.33 ± 0.25*	*0.86 ± 0.11*	*1.78 ± 0.33*
Thalamus	*GM*	*L*	*Sample 1 (Lausanne)*	*0.35 ± 0.03*	*1.50 ± 0.33*	*0.82 ± 0.08*	*1.74 ± 0.25*
Thalamus	*GM*	*L*	*Sample 2 (Geneva)*	*0.35 ± 0.05*	*1.32 ± 0.33*	*0.81 ± 0.12*	*1.75 ± 0.33*
Thalamus	*GM*	*R*	*Sample 1 (Lausanne)*	*0.34 ± 0.02*	*1.58 ± 0.31*	*0.82 ± 0.07*	*1.75 ± 0.30*
Thalamus	*GM*	*R*	*Sample 2 (Geneva)*	*0.35 ± 0.05*	*1.31 ± 0.30*	*0.81 ± 0.12*	*1.75 ± 0.35*
Putamen	*GM*	*L*	*Sample 1 (Lausanne)*	*0.30 ± 0.04*	*1.41 ± 0.36*	*0.76 ± 0.08*	*1.54 ± 0.20*
Putamen	*GM*	*L*	*Sample 2 (Geneva)*	*0.30 ± 0.04*	*1.32 ± 0.35*	*0.76 ± 0.12*	*1.52 ± 0.31*
Putamen	*GM*	*R*	*Sample 1 (Lausanne)*	*0.29 ± 0.02*	*1.40 ± 0.31*	*0.73 ± 0.08*	*1.50 ± 0.27*
Putamen	*GM*	*R*	*Sample 2 (Geneva)*	*0.30 ± 0.04*	*1.29 ± 0.34*	*0.75 ± 0.11*	*1.57 ± 0.34*
Pallidum	*GM*	*L*	*Sample 1 (Lausanne)*	*0.29 ± 0.04*	*1.44 ± 0.46*	*0.72 ± 0.09*	*1.58 ± 0.26*
Pallidum	*GM*	*L*	*Sample 2 (Geneva)*	*0.29 ± 0.04*	*1.23 ± 0.33*	*0.71 ± 0.11*	*1.59 ± 0.34*

Brain region	Type	Side	Cohort	Cho / tCr	Glx / tCr	Ins / tCr	tNAA / tCr
Pallidum	*GM*	*R*	*Sample 1 (Lausanne)*	*0.28 ± 0.03*	*1.40 ± 0.45*	*0.70 ± 0.10*	*1.54 ± 0.28*
Pallidum	*GM*	*R*	*Sample 2 (Geneva)*	*0.29 ± 0.04*	*1.21 ± 0.34*	*0.70 ± 0.11*	*1.62 ± 0.35*
Caudate	*GM*	*L*	*Sample 1 (Lausanne)*	*0.36 ± 0.05*	*1.42 ± 0.34*	*0.89 ± 0.15*	*1.70 ± 0.24*
Caudate	*GM*	*L*	*Sample 2 (Geneva)*	*0.36 ± 0.06*	*1.36 ± 0.39*	*0.82 ± 0.15*	*1.61 ± 0.34*
Caudate	*GM*	*R*	*Sample 1 (Lausanne)*	*0.36 ± 0.04*	*1.47 ± 0.35*	*0.88 ± 0.15*	*1.76 ± 0.24*
Caudate	*GM*	*R*	*Sample 2 (Geneva)*	*0.36 ± 0.08*	*1.30 ± 0.37*	*0.84 ± 0.22*	*1.65 ± 0.38*

Table S4. Cramer-Rao Lower Bound (CRLB) for each metabolite, by brain region

**Brain Region**	**Type**	**Side**	**Cohort**	**CRLB tCr**	**CRLB Cho**	**CRLB Glx**	**CRLB Ins**	**CRLB tNAA**
Frontal	*GM*	*L*	*Sample 1 (Lausanne)*	*4.35 ± 1.20*	*5.53 ± 1.28*	*6.72 ± 2.11*	*5.90 ± 1.48*	*3.76 ± 1.24*
Frontal	*GM*	*L*	*Sample 2 (Geneva)*	*5.49 ± 3.76*	*6.38 ± 3.70*	*8.06 ± 3.30*	*6.64 ± 3.53*	*4.83 ± 3.11*
Frontal	*GM*	*R*	*Sample 1 (Lausanne)*	*4.38 ± 1.09*	*5.51 ± 1.15*	*6.73 ± 2.02*	*5.90 ± 1.34*	*3.80 ± 1.15*
Frontal	*GM*	*R*	*Sample 2 (Geneva)*	*5.71 ± 3.91*	*6.36 ± 2.34*	*8.68 ± 5.33*	*6.70 ± 3.10*	*4.95 ± 3.22*
Frontal	*WM*	*L*	*Sample 1 (Lausanne)*	*3.79 ± 0.76*	*4.69 ± 0.82*	*6.80 ± 1.96*	*5.36 ± 0.98*	*2.99 ± 0.72*
Frontal	*WM*	*L*	*Sample 2 (Geneva)*	*4.62 ± 2.47*	*5.30 ± 1.79*	*8.45 ± 3.34*	*6.14 ± 2.54*	*3.64 ± 1.66*
Frontal	*WM*	*R*	*Sample 1 (Lausanne)*	*3.75 ± 0.71*	*4.65 ± 0.81*	*6.65 ± 1.70*	*5.31 ± 0.92*	*2.98 ± 0.63*
Frontal	*WM*	*R*	*Sample 2 (Geneva)*	*4.73 ± 2.72*	*5.31 ± 1.53*	*8.90 ± 5.29*	*6.19 ± 2.02*	*3.77 ± 1.62*
Parietal	*GM*	*L*	*Sample 1 (Lausanne)*	*3.46 ± 1.18*	*4.87 ± 1.26*	*6.12 ± 2.27*	*5.07 ± 1.46*	*3.10 ± 1.11*
Parietal	*GM*	*L*	*Sample 2 (Geneva)*	*4.24 ± 2.32*	*5.49 ± 1.92*	*7.14 ± 2.66*	*5.69 ± 2.26*	*3.83 ± 2.18*
Parietal	*GM*	*R*	*Sample 1 (Lausanne)*	*3.54 ± 1.04*	*4.93 ± 1.21*	*5.98 ± 1.82*	*5.13 ± 1.35*	*3.19 ± 1.01*
Parietal	*GM*	*R*	*Sample 2 (Geneva)*	*4.41 ± 3.40*	*5.51 ± 1.81*	*7.43 ± 3.97*	*5.89 ± 3.60*	*3.59 ± 1.71*
Parietal	*WM*	*L*	*Sample 1 (Lausanne)*	*3.03 ± 0.80*	*4.22 ± 0.88*	*6.23 ± 1.96*	*4.66 ± 0.99*	*2.48 ± 0.67*

**Brain Region**	**Type**	**Side**	**Cohort**	**CRLB tCr**	**CRLB Cho**	**CRLB Glx**	**CRLB Ins**	**CRLB tNAA**
Parietal	*WM*	*L*	*Sample 2 (Geneva)*	*3.24 ± 0.73*	*4.42 ± 0.85*	*7.33 ± 2.34*	*5.03 ± 1.24*	*2.54 ± 0.65*
Parietal	*WM*	*R*	*Sample 1 (Lausanne)*	*3.08 ± 0.74*	*4.28 ± 0.87*	*6.03 ± 1.43*	*4.65 ± 0.87*	*2.52 ± 0.52*
Parietal	*WM*	*R*	*Sample 2 (Geneva)*	*3.32 ± 0.93*	*4.52 ± 0.97*	*7.52 ± 2.67*	*5.10 ± 1.34*	*2.62 ± 0.72*
Temporal	*GM*	*L*	*Sample 1 (Lausanne)*	*3.58 ± 0.91*	*4.95 ± 1.08*	*6.15 ± 1.60*	*5.25 ± 1.10*	*3.25 ± 0.90*
Temporal	*GM*	*L*	*Sample 2 (Geneva)*	*4.35 ± 3.28*	*5.30 ± 2.13*	*7.21 ± 3.32*	*5.64 ± 2.44*	*3.66 ± 2.62*
Temporal	*GM*	*R*	*Sample 1 (Lausanne)*	*3.77 ± 0.89*	*5.15 ± 1.07*	*6.19 ± 1.43*	*5.31 ± 1.03*	*3.36 ± 0.89*
Temporal	*GM*	*R*	*Sample 2 (Geneva)*	*4.53 ± 2.49*	*5.51 ± 1.73*	*7.81 ± 4.30*	*5.87 ± 2.56*	*3.72 ± 1.90*
Temporal	*WM*	*L*	*Sample 1 (Lausanne)*	*3.40 ± 0.72*	*4.44 ± 0.84*	*6.81 ± 1.47*	*5.09 ± 0.90*	*2.78 ± 0.61*
Temporal	*WM*	*L*	*Sample 2 (Geneva)*	*3.93 ± 1.38*	*4.77 ± 1.23*	*8.28 ± 3.66*	*5.92 ± 2.90*	*3.16 ± 1.07*
Temporal	*WM*	*R*	*Sample 1 (Lausanne)*	*3.47 ± 0.79*	*4.55 ± 0.90*	*6.73 ± 1.27*	*5.08 ± 0.94*	*2.91 ± 0.76*
Temporal	*WM*	*R*	*Sample 2 (Geneva)*	*4.07 ± 1.54*	*4.90 ± 1.36*	*8.24 ± 3.13*	*5.74 ± 1.95*	*3.26 ± 1.27*
Occipital	*GM*	*L*	*Sample 1 (Lausanne)*	*3.91 ± 1.25*	*5.62 ± 1.62*	*6.77 ± 1.80*	*5.59 ± 1.53*	*3.56 ± 1.21*
Occipital	*GM*	*L*	*Sample 2 (Geneva)*	*4.46 ± 3.51*	*6.21 ± 4.09*	*7.76 ± 3.92*	*6.00 ± 3.40*	*3.55 ± 1.74*
Occipital	*GM*	*R*	*Sample 1 (Lausanne)*	*4.00 ± 1.44*	*5.81 ± 1.86*	*6.76 ± 1.93*	*5.61 ± 1.67*	*3.61 ± 1.43*

Brain Region	Type	Side	Cohort	CRLB tCr	CRLB Cho	CRLB Glx	CRLB Ins	CRLB tNAA
Occipital	*GM*	*R*	*Sample 2 (Geneva)*	*4.56 ± 3.25*	*6.22 ± 3.22*	*8.26 ± 4.93*	*6.15 ± 3.37*	*3.95 ± 3.02*
Occipital	*WM*	*L*	*Sample 1 (Lausanne)*	*3.56 ± 0.99*	*4.97 ± 1.28*	*7.00 ± 1.54*	*5.15 ± 1.23*	*2.93 ± 0.83*
Occipital	*WM*	*L*	*Sample 2 (Geneva)*	*4.15 ± 3.86*	*5.55 ± 3.83*	*8.61 ± 5.27*	*5.81 ± 3.43*	*3.12 ± 1.76*
Occipital	*WM*	*R*	*Sample 1 (Lausanne)*	*3.70 ± 1.18*	*5.19 ± 1.45*	*7.03 ± 1.62*	*5.30 ± 1.39*	*3.09 ± 1.13*
Occipital	*WM*	*R*	*Sample 2 (Geneva)*	*3.75 ± 1.42*	*5.04 ± 1.27*	*8.77 ± 6.12*	*5.32 ± 1.71*	*2.99 ± 1.21*
Thalamus	*GM*	*L*	*Sample 1 (Lausanne)*	*4.81 ± 1.08*	*5.08 ± 0.93*	*8.48 ± 2.95*	*6.74 ± 1.10*	*3.87 ± 1.00*
Thalamus	*GM*	*L*	*Sample 2 (Geneva)*	*5.72 ± 10.39*	*6.29 ± 12.84*	*9.83 ± 5.53*	*6.84 ± 4.25*	*3.63 ± 1.55*
Thalamus	*GM*	*R*	*Sample 1 (Lausanne)*	*4.90 ± 1.29*	*5.23 ± 1.23*	*8.27 ± 2.31*	*6.83 ± 1.31*	*4.02 ± 1.26*
Thalamus	*GM*	*R*	*Sample 2 (Geneva)*	*5.27 ± 8.87*	*6.00 ± 10.09*	*9.79 ± 5.06*	*7.10 ± 6.98*	*3.72 ± 2.14*
Putamen	*GM*	*L*	*Sample 1 (Lausanne)*	*4.48 ± 0.87*	*5.16 ± 0.87*	*8.97 ± 3.22*	*6.49 ± 1.12*	*3.80 ± 0.89*
Putamen	*GM*	*L*	*Sample 2 (Geneva)*	*4.85 ± 2.64*	*5.96 ± 3.68*	*9.42 ± 4.55*	*7.14 ± 3.05*	*4.59 ± 3.81*
Putamen	*GM*	*R*	*Sample 1 (Lausanne)*	*4.12 ± 0.72*	*4.93 ± 0.80*	*7.79 ± 2.48*	*6.43 ± 1.07*	*3.43 ± 0.82*
Putamen	*GM*	*R*	*Sample 2 (Geneva)*	*4.59 ± 2.71*	*5.63 ± 2.61*	*9.16 ± 3.23*	*6.76 ± 2.14*	*4.23 ± 2.90*
Pallidum	*GM*	*L*	*Sample 1 (Lausanne)*	*5.13 ± 1.54*	*6.22 ± 1.79*	*10.13 ± 4.10*	*7.72 ± 2.09*	*4.40 ± 1.70*

Brain Region	Type	Side	Cohort	CRLB tCr	CRLB Cho	CRLB Glx	CRLB Ins	CRLB tNAA
Pallidum	*GM*	*L*	*Sample 2 (Geneva)*	*9.51 ± 21.05*	*6.52 ± 3.60*	*10.85 ± 5.79*	*9.13 ± 9.37*	*6.08 ± 11.28*
Pallidum	*GM*	*R*	*Sample 1 (Lausanne)*	*5.28 ± 1.49*	*6.18 ± 1.41*	*9.10 ± 2.83*	*7.60 ± 1.73*	*4.28 ± 1.24*
Pallidum	*GM*	*R*	*Sample 2 (Geneva)*	*6.73 ± 11.15*	*5.99 ± 1.82*	*10.39 ± 3.63*	*7.71 ± 2.27*	*4.70 ± 3.23*
Caudate	*GM*	*L*	*Sample 1 (Lausanne)*	*5.04 ± 1.08*	*5.41 ± 1.12*	*9.22 ± 2.69*	*6.64 ± 1.27*	*3.88 ± 0.93*
Caudate	*GM*	*L*	*Sample 2 (Geneva)*	*7.80 ± 13.17*	*6.14 ± 3.39*	*11.08 ± 5.92*	*8.38 ± 7.04*	*5.90 ± 9.67*
Caudate	*GM*	*R*	*Sample 1 (Lausanne)*	*4.65 ± 1.14*	*4.97 ± 0.99*	*8.25 ± 2.58*	*6.45 ± 1.01*	*3.76 ± 1.01*
Caudate	*GM*	*R*	*Sample 2 (Geneva)*	*6.73 ± 13.58*	*5.77 ± 2.45*	*10.83 ± 7.79*	*7.20 ± 2.79*	*4.52 ± 2.17*

Table S5. Full Width at Half Maximum (FWHM) and Signal-to-Noise Ratio (SNR), by brain region

Brain Region	Type	Side	Cohort	FWHM	SNR
Frontal	*GM*	*L*	*Sample 1 (Lausanne)*	*0.05 ± 0.00*	*15.96 ± 5.48*
Frontal	*GM*	*L*	*Sample 2 (Geneva)*	*0.05 ± 0.01*	*15.88 ± 4.79*
Frontal	*GM*	*R*	*Sample 1 (Lausanne)*	*0.05 ± 0.00*	*15.73 ± 5.30*
Frontal	*GM*	*R*	*Sample 2 (Geneva)*	*0.05 ± 0.01*	*15.36 ± 4.76*
Frontal	*WM*	*L*	*Sample 1 (Lausanne)*	*0.04 ± 0.00*	*19.42 ± 4.72*
Frontal	*WM*	*L*	*Sample 2 (Geneva)*	*0.04 ± 0.00*	*18.88 ± 4.40*
Frontal	*WM*	*R*	*Sample 1 (Lausanne)*	*0.04 ± 0.00*	*19.52 ± 4.50*
Frontal	*WM*	*R*	*Sample 2 (Geneva)*	*0.04 ± 0.00*	*18.65 ± 4.39*
Parietal	*GM*	*L*	*Sample 1 (Lausanne)*	*0.04 ± 0.00*	*21.44 ± 7.23*
Parietal	*GM*	*L*	*Sample 2 (Geneva)*	*0.04 ± 0.00*	*19.99 ± 5.89*
Parietal	*GM*	*R*	*Sample 1 (Lausanne)*	*0.04 ± 0.00*	*20.97 ± 6.79*
Parietal	*GM*	*R*	*Sample 2 (Geneva)*	*0.04 ± 0.00*	*20.09 ± 6.19*
Parietal	*WM*	*L*	*Sample 1 (Lausanne)*	*0.04 ± 0.00*	*24.62 ± 6.34*
Parietal	*WM*	*L*	*Sample 2 (Geneva)*	*0.03 ± 0.00*	*23.69 ± 5.61*
Parietal	*WM*	*R*	*Sample 1 (Lausanne)*	*0.04 ± 0.00*	*24.58 ± 5.96*
Parietal	*WM*	*R*	*Sample 2 (Geneva)*	*0.03 ± 0.00*	*23.59 ± 5.82*
Temporal	*GM*	*L*	*Sample 1 (Lausanne)*	*0.05 ± 0.00*	*15.44 ± 5.01*
Temporal	*GM*	*L*	*Sample 2 (Geneva)*	*0.04 ± 0.01*	*15.62 ± 4.64*
Temporal	*GM*	*R*	*Sample 1 (Lausanne)*	*0.05 ± 0.00*	*14.96 ± 4.46*
Temporal	*GM*	*R*	*Sample 2 (Geneva)*	*0.04 ± 0.01*	*15.18 ± 4.29*
Temporal	*WM*	*L*	*Sample 1 (Lausanne)*	*0.04 ± 0.00*	*18.28 ± 4.62*
Temporal	*WM*	*L*	*Sample 2 (Geneva)*	*0.03 ± 0.00*	*18.21 ± 4.81*

Brain Region	Type	Side	Cohort	FWHM	SNR
Temporal	*WM*	*R*	*Sample 1 (Lausanne)*	*0.04 ± 0.00*	*17.92 ± 4.41*
Temporal	*WM*	*R*	*Sample 2 (Geneva)*	*0.04 ± 0.00*	*17.77 ± 4.49*
Occipital	*GM*	*L*	*Sample 1 (Lausanne)*	*0.04 ± 0.00*	*19.29 ± 7.18*
Occipital	*GM*	*L*	*Sample 2 (Geneva)*	*0.04 ± 0.00*	*19.02 ± 6.22*
Occipital	*GM*	*R*	*Sample 1 (Lausanne)*	*0.04 ± 0.00*	*18.72 ± 7.02*
Occipital	*GM*	*R*	*Sample 2 (Geneva)*	*0.04 ± 0.00*	*19.29 ± 6.02*
Occipital	*WM*	*L*	*Sample 1 (Lausanne)*	*0.04 ± 0.00*	*21.41 ± 6.57*
Occipital	*WM*	*L*	*Sample 2 (Geneva)*	*0.03 ± 0.00*	*21.42 ± 6.42*
Occipital	*WM*	*R*	*Sample 1 (Lausanne)*	*0.04 ± 0.00*	*21.17 ± 6.68*
Occipital	*WM*	*R*	*Sample 2 (Geneva)*	*0.03 ± 0.00*	*21.49 ± 6.19*
Thalamus	*GM*	*L*	*Sample 1 (Lausanne)*	*0.04 ± 0.00*	*14.10 ± 2.82*
Thalamus	*GM*	*L*	*Sample 2 (Geneva)*	*0.04 ± 0.00*	*16.21 ± 4.25*
Thalamus	*GM*	*R*	*Sample 1 (Lausanne)*	*0.04 ± 0.00*	*14.44 ± 3.16*
Thalamus	*GM*	*R*	*Sample 2 (Geneva)*	*0.04 ± 0.00*	*16.04 ± 3.92*
Putamen	*GM*	*L*	*Sample 1 (Lausanne)*	*0.05 ± 0.01*	*13.46 ± 3.03*
Putamen	*GM*	*L*	*Sample 2 (Geneva)*	*0.04 ± 0.01*	*14.29 ± 3.87*
Putamen	*GM*	*R*	*Sample 1 (Lausanne)*	*0.05 ± 0.01*	*14.34 ± 3.76*
Putamen	*GM*	*R*	*Sample 2 (Geneva)*	*0.04 ± 0.01*	*14.68 ± 3.73*
Pallidum	*GM*	*L*	*Sample 1 (Lausanne)*	*0.05 ± 0.01*	*11.65 ± 3.28*
Pallidum	*GM*	*L*	*Sample 2 (Geneva)*	*0.04 ± 0.01*	*12.88 ± 3.84*
Pallidum	*GM*	*R*	*Sample 1 (Lausanne)*	*0.05 ± 0.01*	*12.81 ± 4.48*
Pallidum	*GM*	*R*	*Sample 2 (Geneva)*	*0.04 ± 0.01*	*13.42 ± 4.09*
Caudate	*GM*	*L*	*Sample 1 (Lausanne)*	*0.05 ± 0.01*	*12.58 ± 2.85*
Caudate	*GM*	*L*	*Sample 2 (Geneva)*	*0.04 ± 0.01*	*12.93 ± 3.35*

**Brain Region**	**Type**	**Side**	**Cohort**	**FWHM**	**SNR**
Caudate	*GM*	*R*	*Sample 1 (Lausanne)*	*0.04 ± 0.01*	*13.82 ± 3.43*
Caudate	*GM*	*R*	*Sample 2 (Geneva)*	*0.04 ± 0.01*	*13.32 ± 3.04*

Table S6. MRSinMRS informations

Category	Geneva Study	Lausanne Psychosis Cohort
Scanner	3T Magnetom TrioTim (Siemens)	3T Prisma Fit (Siemens)
RF coils	32 ch 1H head coil	32 ch 1H head coil
Coil elements	HEA;HEP	HEA;HEP
Sequence	3D 1H-FID-MRSI (CS-accelerated)	3D 1H-FID-MRSI (CS-accelerated)
Position	R4.8 A11.8 H36.9	L3.9 A23.0 H4.3
Orientation	T > C-13.5 > S2.5	T > C34.5 > S-5.4
Rotation (deg)	-2	0
TE (ms)	1.5	1.0
TR (ms)	372	353
Averages	1	1
Flip angle (°)	35	40
FOV (mm)	210 × 160 × 105	210 × 160 × 95
Slab thickness (mm)	95	95
Slabs	1	1
Resolution (mm^3)	5 × 5 × 5.3	5 × 5 × 5.3
Spectral bandwidth (Hz)	2000	2000
FID points / Vector size	512	512
Acquisition duration (ms)	256	256
Matrix size	32 × 42 × 20	32 × 42 × 20
Water reference TE (ms)	1.5	1.07

Category	Geneva Study	Lausanne Psychosis Cohort
Water reference TR (ms)	36	25
Water reference flip angle (°)	3	5
Water reference resolution (mm³)	6.6 × 6.7 × 6.6	6.6 × 6.7 × 6.6
Water reference FID points	16	16
Averaging mode	Short term	Short term
Water suppr.	Weak water suppr.	Water sat.
Water suppr. BW (Hz)	60	60
Spectral suppr.	None	None
Measurements	1	1
Saturation bands	2 bands, 20 mm thickness	2 bands, 20 mm thickness
Compressed sensing	acceleration factor 3.3, radius 0.2	acceleration factor 3.3, radius 0.2
Preparation scans	4	4
Dimension	3D	3D
Delta frequency (ppm)	0.00	0.00
Phase encoding	Elliptical	Elliptical
Remove oversampling	On	On
WS timing (ms)	24	22
WS amplitude factor	0.9	0.9
Max gradient amplitude used (mT/m)	26.0	33.00
Shim mode	Advanced	Advanced

Category	Geneva Study	Lausanne Psychosis Cohort
Data processing	Low-rank + TGV reconstruction; lipid/water removal	Low-rank + TGV reconstruction; lipid/water removal
Quantification	LCModel	LCModel
Metabolite basis set (LCModel)	NAA, NAAG, Cr, PCr, GPC, PCh, mI, sI, Glu, Gln, Lac, GABA, GSH, Tau, Asp, Ala	NAA, NAAG, Cr, PCr, GPC, PCh, mI, sI, Glu, Gln, Lac, GABA, GSH, Tau, Asp, Ala
Combined metabolites	tNAA (NAA+NAAG), tCr (Cr+PCr), Cho (GPC+PCh), Ins (mI), Glx (Glu+Gln)	tNAA (NAA+NAAG), tCr (Cr+PCr), Cho (GPC+PCh), Ins (mI), Glx (Glu+Gln)
Quality metrics	SNR, CRLB, FWHM	SNR, CRLB, FWHM

Table S7: Control file of LCModel

```
$LCMODL
sddegz=999.
sddegp= 2.
degzer= 0.00
degppm= 0.00
ppmst= 4.3
ppmend= 1
nunfil= 2048
ndslic= 1
ndrows= 1
ndcols= 1
ltable= 7
lps= 8
lcoord= 9
islice= 1
irowst=1
irowen= 1
icolst= 1
icolen= 1
hzpppm= 123.1887
echot= 1500 (Geneva)| echot= 1 (Lausanne)
dows= T
nsimul= 0
dkntmn = 0.075
$END
```

Table S8: Pre-processing and VBA analyses parameters sum-up

Pre-processing and VBA analyses parameters sum-up		
**Category**	**Parameter**	**Specification**
**Preprocessing**	Software	ANTs (v 2.6.2), PETPVC (v 1.2.12), FSL *bet2* (v6.0.7.7), cat12 (v12.9)
	Modality	Whole-brain metabolic maps (tNAA, Ins, Cho, Glx, tCr)
	Spatial resolution after normalization	$1 \times 1 \times 1\ mm^3$
	PVE correction	Region-based voxel-wise correction (PETPVC toolbox)
	Spike/outlier handling	99th percentile threshold; biharmonic inpainting + 3×3×3 median filtering
	Brain masking	Subject-level Qmask based on SNR >4, FWHM >0.1 or CRLB >20 ; Spikes also added to Qmask
	Registration (MRSI → T1w)	Rigid + SyN (cross-correlation metric), linear interpolation
	Registration (T1w → MNI)	Rigid + affine (mutual information) + SyN (cross-correlation), linear interpolation
	Interpolation order	Linear (for all transformations)
**Statistical model (GLM)**	Software	FSL *randomise* (v6.0.7.7)
	Model type	General linear model (between-group comparisons or correlations)
	Covariates	Age, sex for each analysis
	Group coding	Dummy-coded contrasts with mirror column for group comparison
	Voxel-wise exclusion of low-quality voxels	Integrated via *–mask* and lesion-masking approach (Winkler et al., 2014)
**Permutation testing**	Number of permutations	10,000
	Exchangeability blocks	None (cross-sectional design)
**Inference & correction**	Multiple comparison correction	TFCE (Threshold-Free Cluster Enhancement)
	TFCE parameters	FSL defaults (E=0.5, H=2.0, connectivity=6)
	Significance threshold	$p < 0.05$, family-wise error corrected

	Reporting	Significant clusters displayed in MNI152 space; mean values extracted for post-hoc tests
**Masking / search space**	Gray/white matter restriction	Whole-brain GM and WM (for metabolic VBA)
	WM-only mask (for gFA)	FMRIB58_FA_1mm, threshold 0.25
**Quality control**	Visual QC	Registration and normalization checked for all subjects
	Automated QC thresholds	CRLB < 20; FWHM < 0.1; SNR > 4

Table S9: Coefficient of variation (COV) of tNAA compared to tCr in patients and controls

	**tNAA**	**tCr**	**Difference** (95%CI)	*p-value*
Patients	18.2%	13.7%	0.030-0.058	0.470
Controls	17.3%	15.2%	0.001-0.041	0.494
